# Supplementary material for: Predicting visual function by interpreting a neuronal wiring diagram
Source: Nature. 2024 Oct 2;634(8032):113–23. doi: 10.1038/s41586-024-07953-5 (PMC11446822; doi:10.1038/s41586-024-07953-5)
Supplement: Supplementary file 6 — CRF and ERF predictions for individual TmY4 and TmY9 cells. Analogous to Supplementary Data 3, but for TmY target types. Shown are the top four monosynaptic pathways, the strongest pathway passing through each of the top ten intermediary types (ranking from Extended Data Fig. 7), and the trisynaptic pathway Tm1–TmY–Dm3–TmY (see the section entitled Prediction of spatial normalization). [file 41586_2024_7953_MOESM6_ESM.zip › DataS4/TmY9q/720575940621830326.pdf]

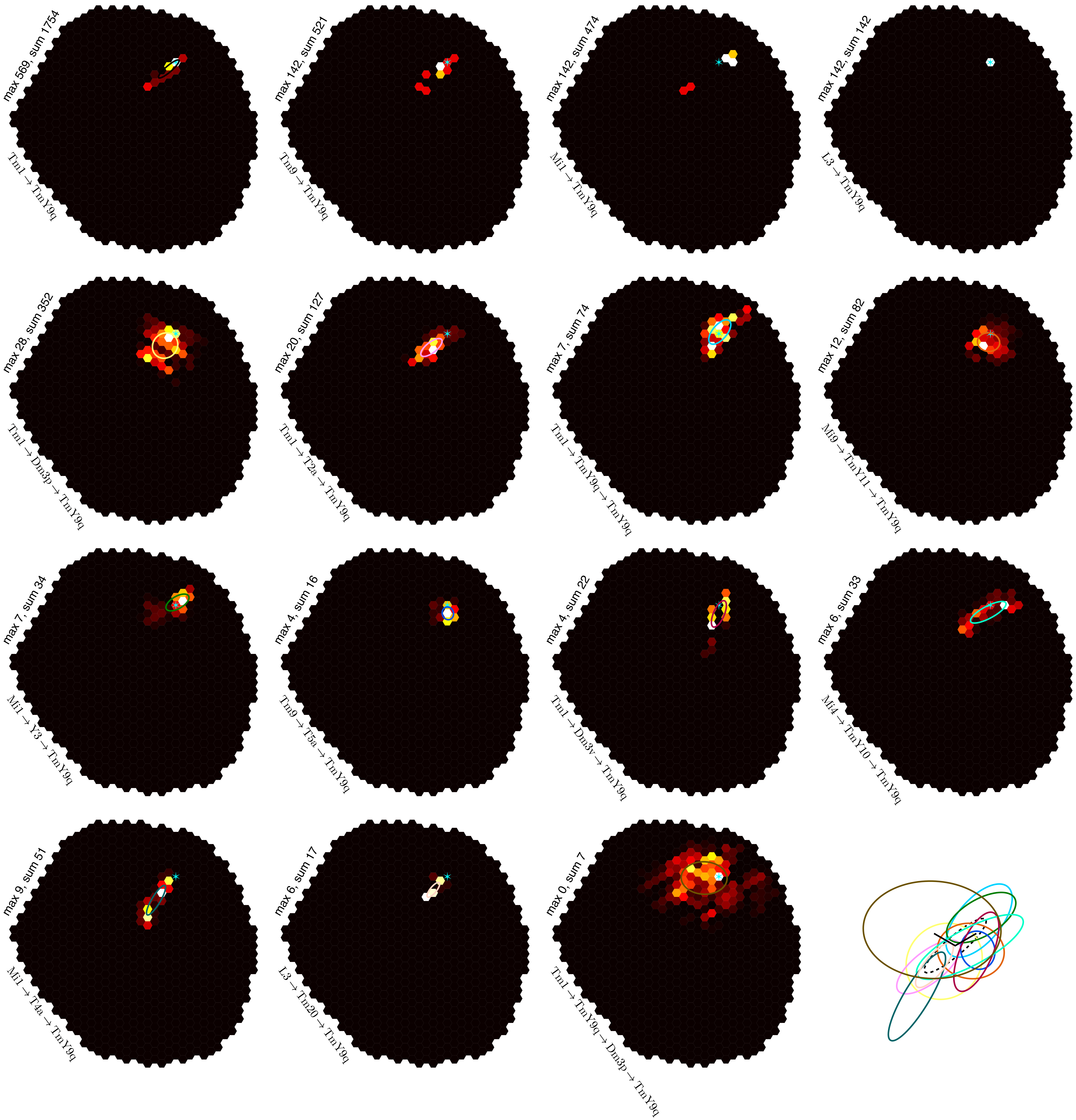
max 569, sum 1754
Tm1 → TmY9q
max 142, sum 521
Tm9 → TmY9q
max 142, sum 474
Mi1 → TmY9q
max 142, sum 142
L3 → TmY9q
max 28, sum 352
Tm1 → Dm3p → TmY9q
max 20, sum 127
Tm1 → T2a → TmY9q
max 7, sum 74
Tm1 → TmY9q → TmY9q
max 12, sum 82
Mi9 → TmY11 → TmY9q
max 7, sum 34
Mi1 → Y3 → TmY9q
max 4, sum 16
Tm9 → T5a → TmY9q
max 4, sum 22
Tm1 → Dm3v → TmY9q
max 6, sum 33
Mi4 → TmY10 → TmY9q
max 9, sum 51
Mi1 → T4a → TmY9q
max 6, sum 17
L3 → Tm20 → TmY9q
max 0, sum 7
Tm1 → TmY9q → Dm3p → TmY9q
